# Contrast quality control for segmentation task based on deep learning models—Application to stroke lesion in CT imaging

**DOI:** 10.3389/fneur.2025.1434334

**Published:** 2025-02-10

**Authors:** Juliette Moreau, Laura Mechtouff, David Rousseau, Omer Faruk Eker, Yves Berthezene, Tae-Hee Cho, Carole Frindel

**Affiliations:** ^1^CarMeN, INSERM U1060, INRAe U1397, Université Lyon 1, INSA de Lyon, Pierre-Bénite, France; ^2^CREATIS, Universite Claude Bernard Lyon 1, INSA Lyon, UMR CNRS 5220, Inserm U1294, Villeurbanne, France; ^3^Department of Neurology, Hospices Civils de Lyon, Bron, France; ^4^LARIS, UMR IRHS INRAe, Universite d'Angers, Angers, France; ^5^Institut Universitaire de France (IUF), Paris, France

**Keywords:** deep learning, segmentation, quality control, contrast analysis, stroke, CT imaging

## Abstract

**Introduction:**

Although medical imaging plays a crucial role in stroke management, machine learning (ML) has been increasingly used in this field, particularly in lesion segmentation. Despite advances in acquisition technologies and segmentation architectures, one of the main challenges of subacute stroke lesion segmentation in computed tomography (CT) imaging is image contrast.

**Methods:**

To address this issue, we propose a method to assess the contrast quality of an image dataset with a ML trained model for segmentation. This method identifies the critical contrast level below which the medical-imaging model fails to learn meaningful content from images. Contrast measurement relies on the Fisher's ratio, estimating how well the stroke lesion is contrasted from the background. The critical contrast is found-thanks to the following three methods: Performance, graphical, and clustering analysis. Defining this threshold improves dataset design and accelerates training by excluding low-contrast images.

**Results:**

Application of this method to brain lesion segmentation in CT imaging highlights a Fisher's ratio threshold value of 0.05, and training validation of a new model without these images confirms this with similar results with only 60% of the training data, resulting in an almost 30% reduction in initial training time. Moreover, the model trained without the low-contrast images performed equally well with all images when tested on another database.

**Discussion:**

This study opens discussion with clinicians concerning the limitations, areas for improvement, and strategies for enhancing datasets and training models. While the methodology was only applied to stroke lesion segmentation in CT images, it has the potential to be adapted to other tasks.

## 1 Introduction

With an estimated 12 million cases each year worldwide, stroke is the second leading cause of death and a major cause of disability (1). Medical imaging is crucial not only for diagnosing and guiding stroke therapy but also for monitoring patients after treatment. The two main imaging modalities for stroke are Magnetic Resonance Imaging (MRI) and Computed Tomography (CT). MRI provides a very accurate image contrast of the brain and the lesion, the latter often provides a lighter or poor image contrast of the brain and the lesion than the surrounding healthy tissue. However, MRI is expensive and requires more time than a CT scan to be performed and is not always available in an emergency. Therefore, in the majority of the cases, CT scan is opted despite its lower contrast; in this modality, the lesion appears darker than the background. The advantage of CT scan is that hemorrhagic transformations are extremely well visible as a light spot in the dark lesion (2).

In recent years, machine learning (ML) has emerged as a powerful tool for segmenting the area of the brain affected by stroke. Despite methodological advances, the subject remains topical, as evidenced by the Ischemic Stroke Lesion Segmentation (ISLES) challenge, which took place annually from 2015 to 2018 and brought together teams from around the world in 2022 (3). As in general medical segmentation, the state-of-the-art architecture in stroke lesion segmentation is the U-Net architecture (4) and its no-new-Net (nnU-Net) variation (5, 6). The nnU-Net, in particular, has led to significant improvements and was the most widely used model in the latest ISLES challenge (7). Reflecting the growing focus on brain lesion segmentation, many alternative architectures have been proposed, utilizing both two-dimensional (2D) and three-dimensional (3D) models and a variety of deep learning techniques. For instance, 3D neural network architectures have been explored in a previous study (8), while other studies leverage multiple imaging modalities (9). Additionally, for improved segmentation accuracy, some studies adopt innovative approaches such as generative adversarial networks (GANs) (10) or employ vision transformers (VIT) (11).

Despite continuous advancements in stroke lesion segmentation, applying ML techniques to CT images remains a significant challenge. Due to the limited availability of large annotated public CT datasets until 2023, majority of the existing research has focused on MRI because of the difficulty that represents this imaging modality. Those inherent difficulties of CT imaging, such as lower contrast resolution compared to MRI, pose unique obstacles for stroke lesion detection. As an example, in a recent brain lesion segmentation challenge on joint segmentation of CT scan or MRI lesions (12), the top-performing team achieved a DSC of 0.67 on MRI, but only 0.20 on CT using the same acute stoke patients' tests. One critical factor is the timing of CT image acquisition, as lesion visibility can change dramatically in the first few hours and days after a stroke (13). Our focus on subacute CT images acquired 24 h after the stroke addresses a crucial window for stroke treatment, yet this phase remains underexplored in current research.

A closer examination of existing CT stroke lesion segmentation datasets reveals several additional challenges. Many datasets consist only of follow-up images, and the populations studied often do not reflect the diverse clinical characteristics of stroke patients. Moreover, much of the research has concentrated on hemorrhagic lesions (14, 15), which are easier to detect on CT due to their hyperdense appearance. Other studies exhibit biases in lesion size; for instance, in one study (16), the median lesion volume in the training set was 48 ml (using follow-up images), while the median lesion volume for MRI in the ISLES 2015 challenge was only 17 ml. However, lesion contrast—rather than size alone—appears to be the more significant challenge (17), as ischemic stroke lesions are often poorly contrasted with surrounding healthy tissue in CT images, making them difficult to distinguish.

Addressing these challenges requires specialized approaches for CT analysis. Techniques, such as window setting, which adjusts the Hounsfield unit (HU) ranges to enhance lesion visibility, have shown potential for improving CT segmentation (18). However, often, these methods fail when distinguishing ischemic lesions from normal tissue, as their intensity ranges can overlap significantly. Moreover, there is no consistent HU threshold that can reliably differentiate between healthy tissue and various types of brain lesions (19). To overcome these obstacles, innovative solutions are needed that account for the unique characteristics of CT imaging and the complexities of stroke lesion segmentation, particularly for subacute ischemic lesions, where lesion contrast and visibility, especially, are challenging.

The contrast issue is well-known among medical images analysis and various techniques have been developed to improve it, including morphological transformations (20) and super-resolution (21). Segmentation algorithms have specifically targeted low-contrast images with attention mechanisms on the objects of interest (22–24) or their contours (25). Although, these methods improve segmentation in specific scenarios, they are generally task-specific and they lack generalizability across other medical imaging challenges. Moreover, automatic quality assessment methods for CT datasets primarily evaluate acquisition-related factors (26), such as motion artifacts or field of view, or the quality of specific image processing steps such as registration (27). However, they do not address how the quality of a dataset impacts its suitability for specific machine learning tasks, particularly segmentation.

We aimed to develop a method for assessing the quality of CT datasets, specifically addressing the challenges posed by low contrast in CT scans. Unlike existing methods, our approach evaluates dataset quality in the context of a specific ML task. Our goal is to determine the minimum contrast value required for an ML model to successfully segment a stroke lesion on CT images. By exploring the constraints of low-contrast data, we seek to deepen the understanding of how contrast impacts segmentation performance. To achieve this, we developed a methodology based on performance, graphical, and clustering analysis that can be applied to any medical segmentation task using deep learning architectures. Our approach automates the quality control process, enabling efficient training of segmentation models by ensuring that only the most relevant data is used. This study provides a new tool for assessing dataset quality and addresses one of the main obstacles to CT segmentation of brain lesions.

## 2 Material and methods

### 2.1 Material

We used two independent databases, one for training the ML model and other for testing, to guarantee the generalizability of our results.

#### 2.1.1 Training data: HIBISCUS-STROKE dataset

We included patients from the HIBISCUS-STROKE cohort. The design and methods of this cohort have been published in Debs et al. (28). Briefly, stroke patients with an anterior circulation stroke threated by thrombectomy underwent a baseline MRI and both CT and MRI follow-up scans, respectively, 24 h and 6 days after the treatment. This day-6 MRI is segmented to have a final lesion mask. The work is based on the hypothesis that the lesion is stabilized 24 h after treatment, and so the segmentation obtained on fluid-attenuated inversion recovery (FLAIR MRI) is used as reference for the CT scan.

A total of 108 patients are included in the study. All patients gave their informed consent and the imaging protocol was approved by the regional ethics committee.

#### 2.1.2 Test data: RELATE dataset and APIS dataset

RELATE is a monocentric prospective database of all consecutive patients treated by intravenous thrombolysis and/or thrombectomy, from the same center as for the HIBISCUS-STROKE cohort. The patients were selected with additional criteria compared to the HISBISCUS-STROKE dataset, specifically considering stroke severity scores: Alberta Stroke Program Early Computed Tomography Score (ASPECTS) < 6 and National Institutes of Health Stroke Scale (NIHSS) >7, resulting in the inclusion of only more severe patients. In these RELATE patients, the final stroke lesion was segmented on day 1 CT images, with an additional selection based on the ability of the annotators to accurately segment the images due to the difficulty that the manual CT scans segmentation represents, as day-6 MRI is not part of standard clinical practice. In total, 125 patients were included from this dataset. Due to the selection bias resulting from the severity scores and the ability of the annotators to accurately segment the images, the fact that the reference does not come from the same modality and that the RELATE dataset does not provide day-6 MRI, we could not fuse the two datasets, even though the images come from the same center.

A Paired CT-MRI Dataset for Ischemic Stroke Segmentation (APIS) Challenge, is the only stroke lesion segmentation challenge based on CT images (12). The dataset includes CT images from 60 patients in the acute phase, along with corresponding lesion masks derived from acute-phase ADC MRI scans. Since our work focuses on subacute images, we did not merge this dataset with ours. However, we still found it valuable to use the APIS dataset for testing to assess how our models perform on a different set of images.

#### 2.1.3 Preprocessing

Before training and testing the models, some preprocessing steps specific to stroke lesion segmentation task were performed: (1) skull stripping with FMRIB Software Library (FSL), optimized for CT images (29, 30); (2) non-linear registration of images onto DWI MRI as a reference frame using ANTs (31); (3) separation of 3D volumes into 2D slices, with only slices containing lesions according to the reference being retained; (4) selection of slices containing lesions with an area >1cm^2^, as smaller lesions cannot be reliably segmented on CT; (5) selective horizontal flipping to place all lesions in the same hemisphere and provide a consistent lesion position prior; and (6) resizing of images to 192 × 192 pixels. Steps 3 and 4 assume that a rough location of the lesion is known from preliminary clinical examinations. Ultimately, the 108 patients included in the HIBISCUS-STROKE dataset represent 3,772 slices used for training the model. Treated the same way, the patients in the RELATE database represent 4,327 samples, and the APIS database contains 259 images, on which models are tested to test their transferability.

### 2.2 Methods

**Figure 1:**
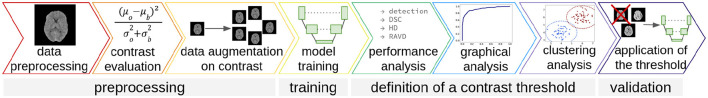
Graphical representation of the methodology steps. The performance analysis are performed on results of models trained on raw dataset or dataset with data augmentation.

Using the HIBISCUS-STROKE database, we identified the minimal image contrast for an ML model to successfully segment a subacute stroke lesion. The proposed method is summarized in Figure 1 and detailed in the following subsections.

#### 2.2.1 Contrast evaluation

The key element of our study is the evaluation of the contrast of the stroke lesion in the CT images. We used Fisher's ratio that is well-adapted to evaluate the contrast between an object of interest and the background. It is defined as follows:


$$\begin{array}{l} F=\frac{{\left(\mu_{object}-\mu_{background}\right)}^{2}}{\sigma_{object}^{2}+\sigma_{background}^{2}} \end{array}$$

where μ_*object*_ represents the mean of the values within the object of interest according to a ground truth, μ_*background*_ represents the mean of the values in the background tissue, and $\sigma_{object}^{2}$ and $\sigma_{background}^{2}$ are the standard deviations squared in these respective regions. The Fisher's ratio assumes normally distributed data within each group and approximately equal variances across different groups.

#### 2.2.2 Data augmentation

The training dataset underwent data augmentation (DA) to balance the distribution based on CT image contrast. This augmentation process occurs in two steps, each tailored to address specific aspects of the image data.

Initially, the lesion pixel density were systematically reduced by two HU, iteratively over three rounds, while preserving a border row of pixels unchanged to prevent the emergence of artifacts. This progressive reduction aims to create a smooth gradient within the lesion and mitigate the appearance of outlines resulting from drastic value modifications.

In a second step, applied selectively to slices exhibiting a higher standard deviation of lesion intensity than the background, further modifications were made to pixels falling below or above the median of the lesion's pixel values. Specifically, a modification of two units was applied to these pixels to equalize the appearance of inhomogeneous lesions. Subsequently, a selection process guided by the Fisher's ratio of the augmented images was employed, ensuring that the contrast remains within the range observed in the original dataset. Slices with overly low contrast were excluded, as they were already well-represented in the dataset.

For a detailed outline of this heuristic process, Supplementary Algorithm 1 provides a concise pseudocode overview.

#### 2.2.3 Model training and testing

The U-Net (4) architecture was used to segment the stroke lesions in the CT images. It consists of a contractive path with 3*3 convolutional layers followed by a rectified linear unit (ReLU) activation function (32) and a 2 × max pooling operation. On the other side, there is an expansive path including an upsampling of the feature map, followed by a 2 × 2 convolution and concatenation with the corresponding feature map from the contractive path called skip connection, and two 3 × 3 convolutions, each followed by a ReLU. The training was done with Adam optimizer (33) and the sum of the Dice loss, based on the Dice coefficient, and the binary cross-entropy:


$$\begin{array}{l} L=\frac{2\times y\times p}{y+p}-\left(y\log\left(p\right)+\left(1-y\right)\log\left(1-p\right)\right) \end{array}$$

where *y* is the ground truth and *p* is the prediction made by the model.

The U-net parameters were optimized by grid search, which consists of training several models with different sets of hyperparameters and selecting the one that performed best on the validation data. The parameters of the optimizer were thus set to β_1_ = 0.5 and β_2_ = 0.999 and the learning rate at *lr* = 0.0005. For the final models with both raw data and DA, a five-folds cross validation was performed. A maximum of 200 epochs were done and early-stopping was applied to the validation set to avoid overfitting. Only around 30 epochs were needed to complete model convergence. The final performances were computed from the test set.

**Figure 2:**
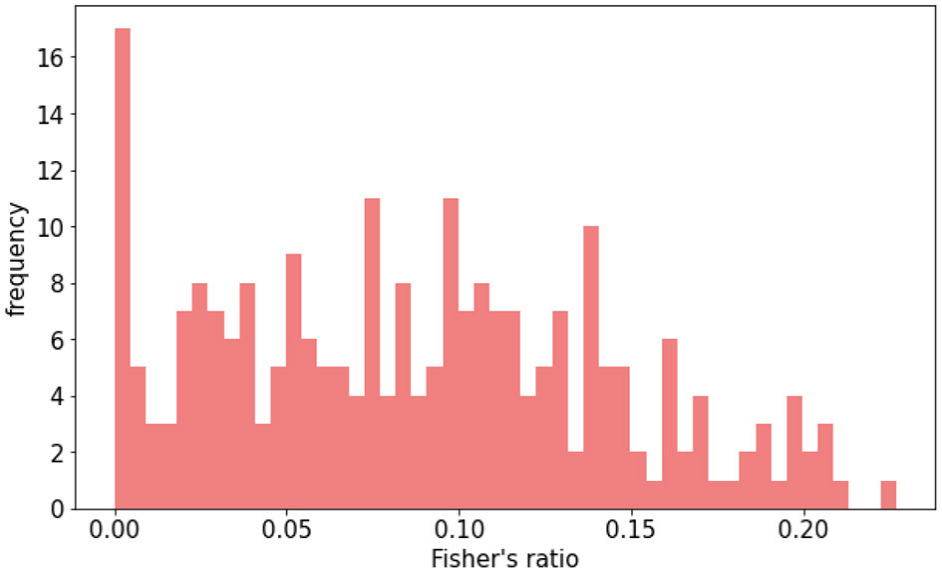
Distribution of Fisher's ratio in HIBISCUS-STROKE test set. Patients are selected, so the distribution is uniform in order to be able to compare the performance between different contrast ranges.

The models were tested on a separate group of patients, who were not used during training. Only 5% of the patients in the original dataset were selected for testing. This selection process operates at patient level, preserving the independence of the test set. Data augmentation (DA) was deployed on the remaining 95% of patients, representing 3,527 training slices. With DA, this number rose to 14,415 slices, while 245 slices were reserved for testing without generating augmented data. The test subset was deliberately balanced to ensure a fair representation of slices across all contrast ranges, as shown in Figure 2. By comparing models trained with and without data augmentation, we checked whether uneven distribution influences poor performance in specific contrast scenarios.

#### 2.2.4 Evaluation metrics

Four evaluation metrics were used to assess segmentation performance:

- Detection, determines if a lesion is present in a slice. It is binary, where 0 denotes the model's failure to segment a lesion where one exists according to the reference, and 1 signifies successful segmentation.
- Dice similarity coefficient (DSC), represents the superposition of the segmentation made by the model and the reference:

$$\begin{array}{l} DSC=\frac{2\times TP}{FN+FP+2\times TP} \end{array}$$

where *TP* are the true positives, *FN* the false negatives, and *FP* the false positives. Unsatisfactory results yield a DSC of 0. A value below 0.1 (10% overlap) is deemed unacceptable, although this threshold may vary depending on the task.

- Hausdorff distance (HD) between the segmentation (A) and the ground truth (B) is expressed as

$$\begin{array}{r} HD=max\left(h\left(A,B\right),h\left(B,A\right)\right)\;\text{where}\\ \;h\left(A,B\right)=\underset{a\in A,b\in B}{maxmin}||A-B||. \end{array}$$

It calculates maximum distance, in pixels, between segmentation and ground truth. With images of size 192 × 192 pixels and the brain typically occupying 70% of the slice height, a threshold of 60 pixels was set to prevent erroneous predictions in different brain regions (e.g., frontal instead of occipital).

- Relative Absolute Area Difference (RAAD) calculated by

$$\begin{array}{l} RAAD=\frac{\left(TP+FP\right)-\left(TP+FN\right)}{TP+FN}. \end{array}$$

It compares segmentation area to ground truth, normalized by the latter's area. The optimal value is 0. The RAAD value exceeding –0.5 or 0.5 indicates significant underestimation or overestimation, respectively, rendering the segmentation unsatisfactory.

Establishing satisfactory metric thresholds aided in identifying initial contrast thresholds in model performance analysis before proceeding with further evaluations.

#### 2.2.5 Contrast threshold definition

To gain an initial estimate of the contrast threshold value, we analyzed the model's performance using the performance thresholds outlined in Section 2.2.4, specifically in relation to contrast. This approach allowed us to categorize the samples based on their contrast, identifying those that perform well or poorly, and enabling the initial determination of the critical Fisher's ratio.

The first method for assessing the contrast threshold in relation to the model's performance involved *graphical analysis* using a receiver operating characteristic (ROC) curve. This curve plots the true positive rate (TPR) against the false positive rate (FPR), for different thresholds of Fisher's ratio. These two rates are calculated as follows:


$$\begin{array}{l} TPR=\frac{TP}{TP+FN}\;FPR=\frac{FP}{FP+TN}. \end{array}$$

This approach involves calculating both TPR and FPR using different test sets, while gradually tightening the contrast threshold. By considering all slices, the TPR might be lower compared to when only images with good contrast are considered. In this type of plot, a distinct “elbow” is typically observed in the upper left corner of the curve, which denotes the best compromise between the TPR and FPR, allowing us to detect the best contrast threshold, related to this best compromise, can be identified at the point closest to this elbow. This point ensures a sufficiently high TPR while maintaining acceptable performance.

The second method relied on *clustering analysis* to identify two distinct groups of slices with varying contrast based on their segmentation performance. The aim is to separate slices that are easy to segment from those that are more difficult. This analysis uses two unsupervised clustering algorithms: the k-means algorithm (34) and the hierarchical clustering algorithm (35). The evaluation metrics (as presented in Section 2.2.4) and Fisher's ratio are used as features after being normalized between 0 and 1 for the clustering algorithms. To determine if there are performance differences correlated to contrast, the resulting centroids of each cluster are compared. The first algorithm, the k-means algorithm, is an iterative process where random cluster centers are assigned, data points are allocated to the closest cluster based on distances, new cluster centers are recalculated, and the process is repeated until stabilization. To mitigate the impact of random initialization, this is repeated 10 times. In contrast, the hierarchical clustering algorithm initially treats each data point as a separate cluster and progressively merges the closest clusters until only two clusters remain. Both algorithms are tested with various distance metrics, such as Euclidean distance (36), squared Euclidean distance (37), Manhattan distance (36), Chebyshev distance (38), and Canberra distance (36). The less variation there is between methods and distances, the more confident the separation is. To determine the best method and distance, two quality measures are used: the purity and silhouette scores. The purity score indicates the percentage of accurately classified objects according to a predefined Fisher's ratio value, while the silhouette score measures the difference between the cohesion within clusters and the distance between clusters. A higher silhouette score, closer to 1, indicates better performance. Since the first method is supervised by a Fisher's ratio threshold and the second is unsupervised, we can assess their correlation. If they are well-correlated, it would confirm the consistency of the selected Fisher's ratio threshold. Additionally, we can vary the threshold used to calculate the purity score to find the value that best correlates with the silhouette score.

#### 2.2.6 Contrast threshold validation and model transferability

To validate the contrast threshold found with graphical and clustering methods, we trained a model removing the slices with a Fisher's ratio under this threshold and compared its performances with the one trained with the whole dataset. If the performances are steady even if some data is removed, it means that the removed data did not bring informative knowledge concerning the segmentation task, enabling us to conclude on the critical contrast threshold. Furthermore, this allows for quantifying potential computational cost reduction based on the extent of data removal during the training phase.

Since we possess additional datasets for stroke lesion segmentation *via* CT scan, it presents an opportunity to assess the applicability of models trained in the preceding section on the RELATE and APIS datasets. This evaluation aims to determine whether these models can be directly transferred to another database, despite being trained without low-contrast slices. If the outcomes align closely, it reinforces the validity of the threshold, indicating that low-contrast images do not contribute significant information during training, even when the models are deployed on a novel dataset.

## 3 Results

All the pipeline being explained, we can apply it to the HIBISCUS-STROKE dataset to determine the contrast threshold for stroke lesion segmentation on CT scan, to control the quality of the dataset for this task with respect to the 2D U-Net model.

### 3.1 Image contrast in the two datasets

As reported in a previous study, we observed a Gaussian distribution of HU values in CT data (39). We assessed normality through histogram analysis and homogeneity *via* variance comparisons using dispersion plots. This allowed us to employ Fisher's ratio to quantify lesion contrast in our images. Fisher's ratio is calculated for each slice after applying a threshold to the HU of the scanner: pixels with values >80 were eliminated to avoid including the skull, while pixels with values < 15 were also discarded or exclude cerebrospinal fluid and calcifications, as had been similarly implemented in previous studies (40, 41). Subsequently, the slices were normalized between 0 and 1. Given that the lesion is the object of interest, the mean and standard deviation values used in Fisher's ratio calculations corresponded to the HU of the lesion and the HU of the healthy tissue in the ipsilateral hemisphere.

**Figure 3:**
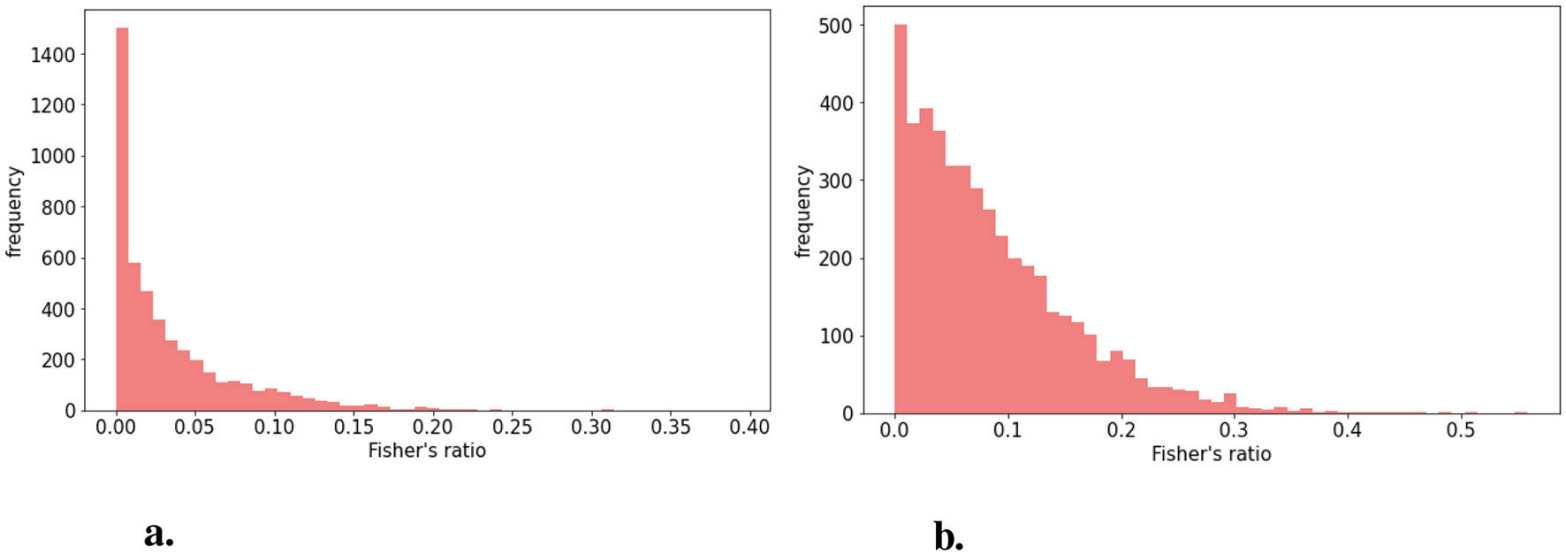
Distribution of Fisher's **(A)** in the HIBISCUS-STROKE dataset which is highly imbalanced **(B)** in the RELATE dataset which is less imbalanced and with higher maximum value.

**Figure 4:**
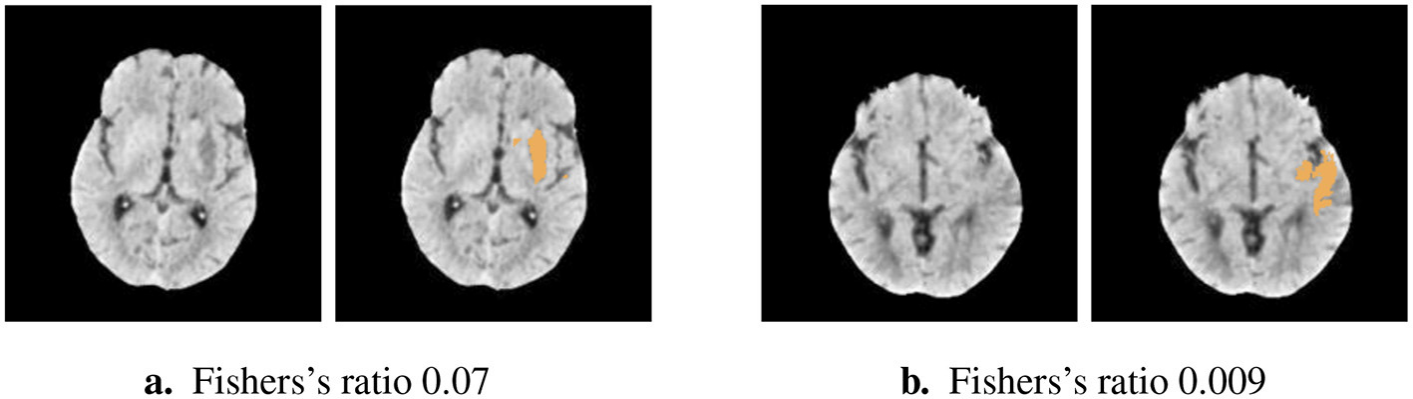
Examples of slices of different lesion contrast with their reference **(A)** higher contrast **(B)** lower contrast showing the variability among the dataset and the difficulty of segmentation of stroke lesion segmentation on CT scans.

The distribution of Fisher's ratio in the HIBISCUS-STROKE dataset (Figure 3A) revealed a significant imbalance, with a predominance of very low contrast slices, expectedly the most challenging to segment. Examples of such slices are depicted in Figure 4, highlighting the considerable contrast variability evident in CT scans, underscoring the difficulty of the segmentation task.

Similarly, Fisher's ratio was computed for the RELATE dataset, resulting in the distribution depicted in Figure 3B.

### 3.2 Raw dataset vs. data augmentation

**Figure 5:**
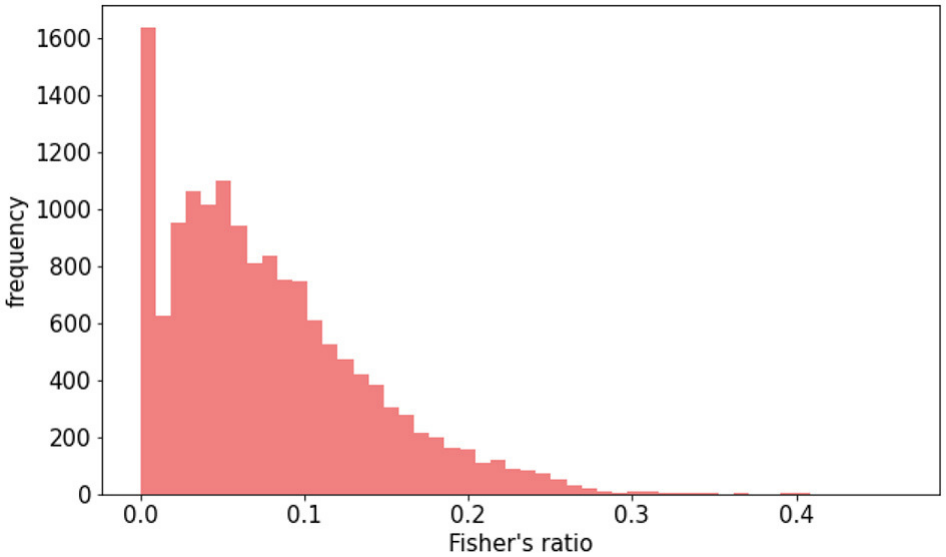
Distribution of Fisher's ratio in augmented HIBISCUS-STROKE dataset in which the imbalance is reduced.

To examine how imbalances in lesion contrast within the dataset affected model performance, we initially compared models trained on raw data with those trained using DA. The Fisher's ratio distribution in the DA dataset is represented in Figure 5. Results outlined in Table 1 indicate that DA consistently enhanced all performance metrics.

**Table 1:** Quantitative results of models trained with or without DA.

	**Detection rate**	**DSC**	**HD**	**RAAD**
Raw	94 ± 3	0.58 ± 0.02	21 ± 1	0.23 ± 0.12
DA	95 ± 1*	0.65 ± 0.03***	19 ± 2**	−0.06 ± 0.04***

One-tailed unpaired Mann–Whitney statistical test if a metric is significantly better for a dataset than another (*** if *p* < 0.01, ** if *p* < 0.05, * if *p* < 0.09).

**Figure 6:**
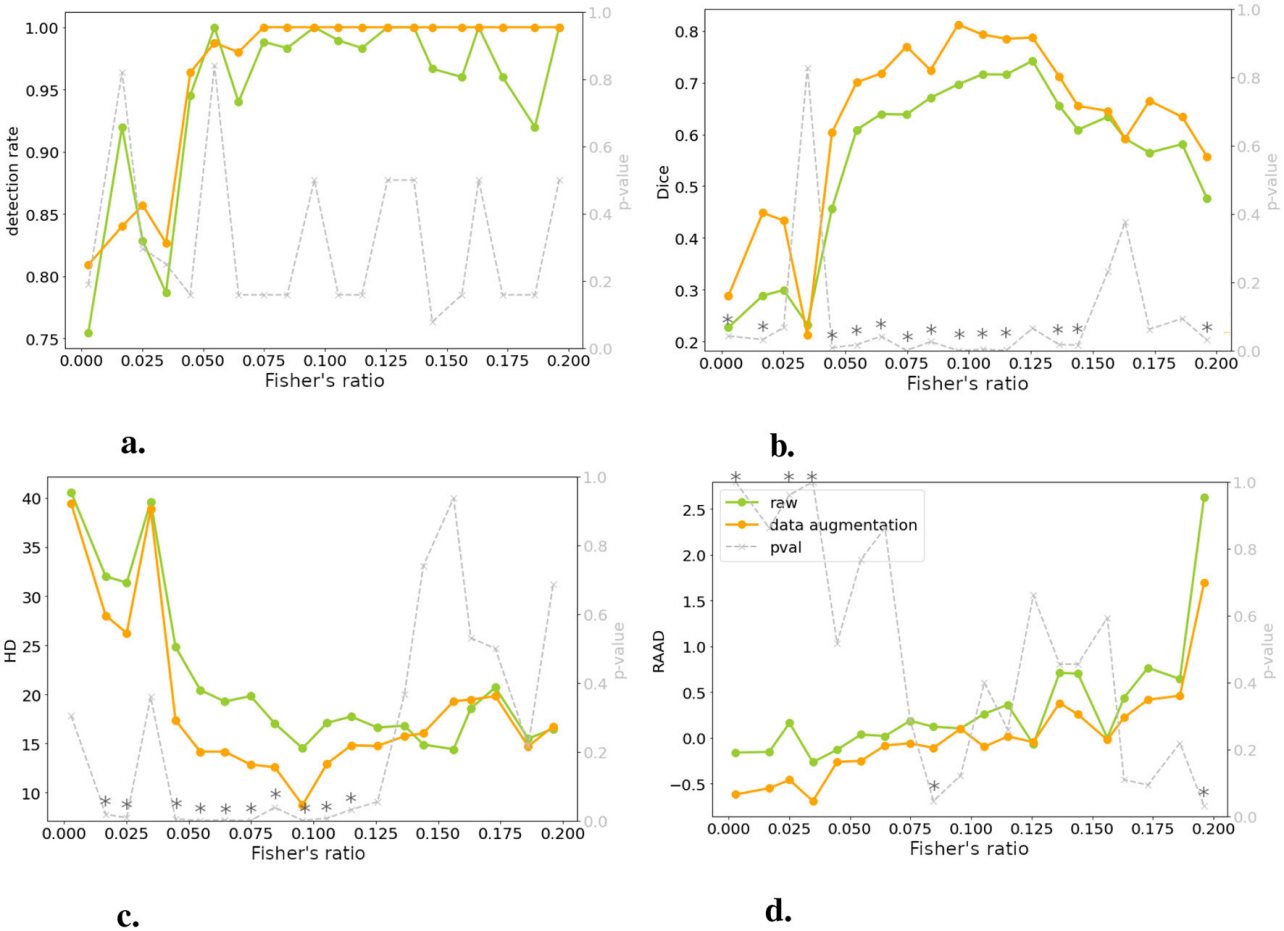
Comparing models trained on raw and DA data for four metrics: performance is plotted against test set image contrast. One-tailed Wilcoxon tests determine if DA-trained model outperforms raw-trained model (gray dotted line, right *y*-axis; * indicates *p* < 0.05 or > 0.95). There is no significant differences between raw and DA for a specific range of contrast, meaning that the contraste imbalance does not penalize a specific range of contrast. **(A)** Detection rate. **(B)** DSC. **(C)** HD. **(D)** RAAD.

A more detailed analysis, correlating performance metrics with Fisher's ratio (Figure 6), revealed that models trained with DA consistently outperformed those trained solely on raw data. Notably, this improvement was most pronounced for intermediate contrast values, which aligned with the focus of our DA strategy. Therefore, we decided to consider exclusively the models with DA for the rest of the study.

### 3.3 Performance in relation to contrast

Figure 6 demonstrated a decline in lesion detection rate, DSC, and HD for slices with a Fisher's ratio below 0.05. Notably, 82% of the undetected lesions in both models (with and without DA) exhibited a Fisher's ratio below 0.05. Similarly, when considering a DSC threshold of 0.1 (as defined in Section 2.2.4), 77% of slices with unsatisfactory DSC had a Fisher's ratio below 0.05. When examining the HD metric, this proportion increased to 100%. Interpreting RAAD was more complex due to two thresholds concerning underestimation and overestimation of the lesion. When focusing on RAAD values below –0.05, 79% of the slices demonstrated a contrast below the threshold, while overestimation of the lesion area was less prevalent. Generally, the second case (without DA) displayed fewer instances of overestimated slices, as discussed in Section 3.2.

### 3.4 Graphical analysis

**Figure 7:**
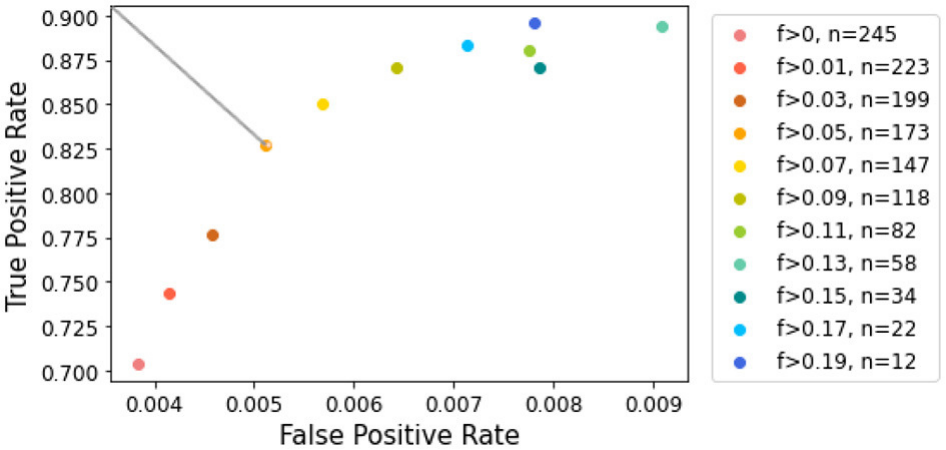
ROC curve for lesion detection given contrast values. The color scale corresponds to contrast values increasing in magnitude. The top left point corresponds to a contrast of 0.05, which allows the most significant gain in true positive rate.

To assess performance at different contrast levels, we employed ROC curve analysis, measuring false positive rate (FPR) and true positive rate (TPR) using various contrast thresholds. Figure 7 illustrates the resulting FPR and TPR, with each point representing a distinct contrast threshold. It may appear counterintuitive that the FPR increases with contrast, as higher contrast is generally associated with easier segmentation. However, Figure 7 reveals that the *x*-axis labels were significantly compressed compared to the *y*-axis. It is important to note that despite the increase, the FPR remained consistently below 1% for all contrast levels. Moreover, as discussed in Section 3.3, when contrast is very low, lesions tend to be underestimated, which can lead to a lower FPR. Conversely, as the contrast increases, the trend reverses, indicating a possible increase in false positives.

When examining the axis scales, particular attention should be given to the TPR. Our analysis showed that removing slices with the lowest contrast has a greater impact on improving TPR during the early stages of elimination. However, beyond a Fisher's ratio of 0.05, further increases in TPR became less noticed. This threshold provided the best balance, retaining a reasonable number of slices while ensuring accurate lesion estimation. Focusing on the cropped ROC curve shown in Figure 7 [following the AUCReshaping method described in Bhat et al. (42)], we observed that the point corresponding to the exclusion of slices with a Fisher's ratio below 0.05 is closest to the top-left corner. This point represents the optimal compromise between TPR and FPR in this range.

### 3.5 Clustering approach

To further corroborate the Fisher's ratio threshold of 0.05, we used unsupervised clustering methods to determine whether distinct groups could be identified according to lesion contrast.

**Figure 8:**
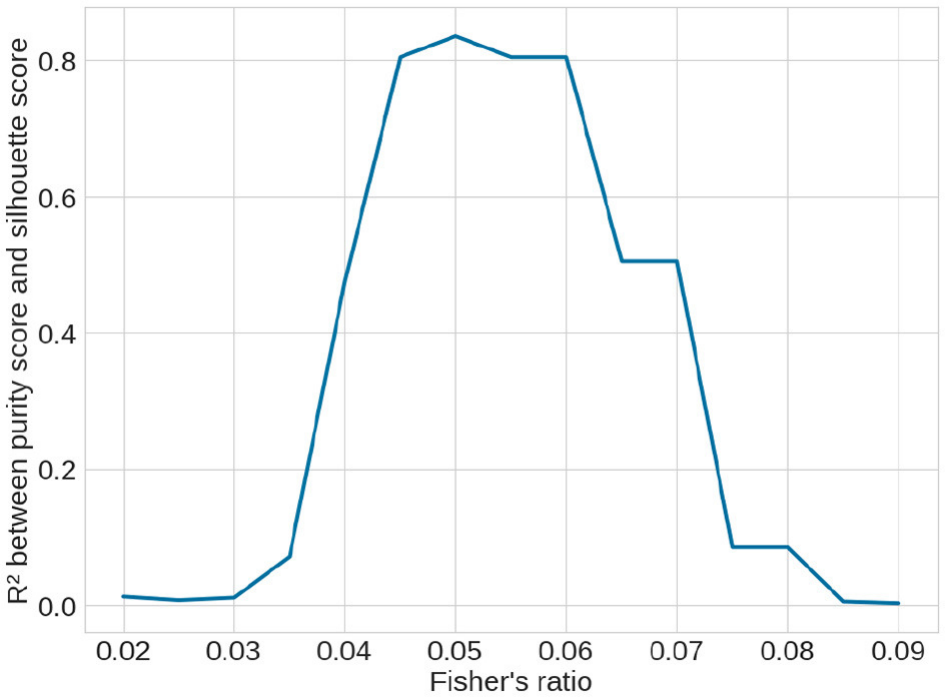
Variation of *R*^2^ correlation coefficient between the unsupervised silhouette score and the supervised purity score over the clustering methods (see Table 2) depending on the contrast threshold selected to supervise the purity score. The best coefficient is obtained with a threshold at 0.05, showing that it is the best threshold.

The quality scores of the clusters, defined in Section 2.2.5, are presented in Table 2. According to the silhouette score, k-means clustering with the Canberra distance yielded the best results. The purity score, which is based on predefined clusters (slices with a Fisher's ratio below or above 0.05), also showed its highest value with k-means using the Canberra distance. This provided an initial validation of the clusters. The correlation between the silhouette score and the purity score is 0.804, further confirming that a Fisher's ratio threshold of 0.05 effectively separated slices based on performance metrics. When considering the variability among the methods, the standard deviation across all purity scores was 0.5%, while it was 0.013 for the silhouette score, accounting for ~0.7% of all possible values. These values indicated the successful separation of data points into two clusters. To further validate the threshold, we examined the correlation between the silhouette score and the purity score across different contrast threshold values. The variation in the *R*^2^ coefficient, as shown in Figure 8, demonstrated that the best correlation is achieved with a threshold of 0.05.

**Table 2:** Clustering quality scores according to the different methods and metrics used.

**Algorithm**	**Metric**	**Silhouette score**	**Purity score (%)**
k-Means	Euclidian	0.597	80.8
k-Means	Squared euclidian	0.597	80.8
k-Means	Manhattan	0.600	81.2
k-Means	Chebyshev	0.552	79.6
k-Means	Canberra	0.601	**81.6**
HC	Euclidian	0.587	80.8
HC	Squared euclidian	0.597	80.8
HC	Canberra	**0.603**	80.8

Purity scores are based on a threshold of 0.05. Bold values indicate best scores.

**Figure 9:**
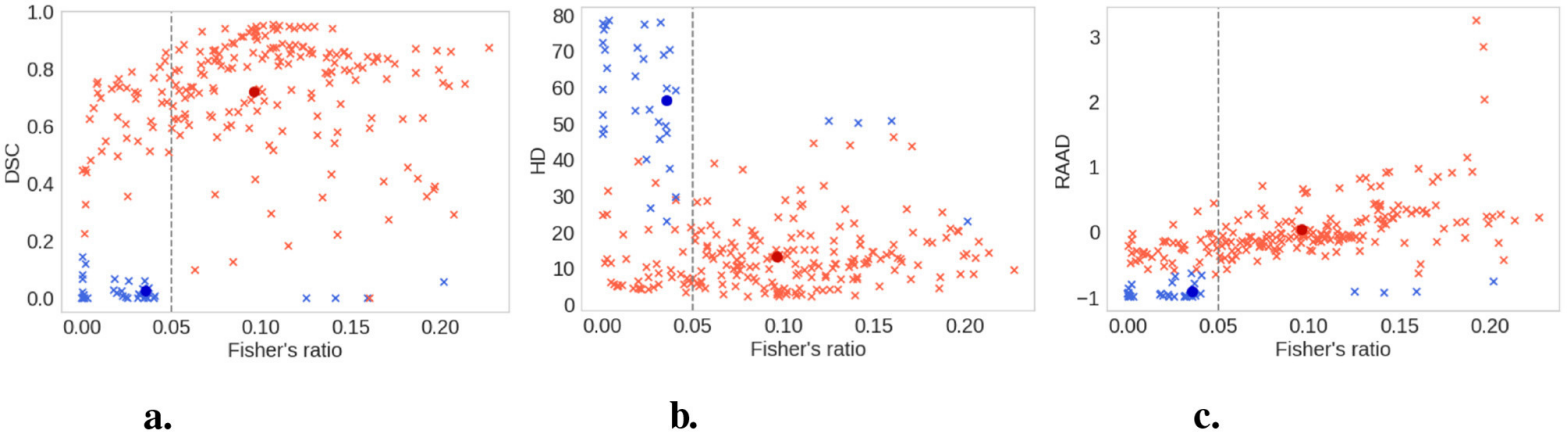
Clusters (good contrast in red and low contrast in blue) with the k-means method and the Canberra distance. All slices are the light crosses, the centroids of the clusters are the dark dots. Vertical dashed line represents Fisher's ratio threshold. The 0.05 threshold is the best value to separate the clusters created by the clustering method. **(A)** DSC. **(B)** HD. **(C)** RAAD.

After examining the clusters shown in Figure 9, it was apparent that the separation based on the Fisher's ratio of 0.05 was not as distinct as anticipated. However, the centroids of the clusters were clearly discernible. The Fisher's ratio means for the two clusters were 0.039 and 0.101, respectively. Nevertheless, there were still some slices with low contrast that were classified alongside well-segmented slices with high contrast. Fortunately, when evaluating the DSC or the HD, these slices exhibited a satisfactory segmentation outcome.

### 3.6 Validation

To validate the defined contrast threshold, we determined whether the slices with low contrast provided useful information. We trained a new model using the same parameters and dataset as in Section 2.2.3, excluding any slices with a Fisher's ratio below 0.05. The hyperparameters were optimized with grid search as detailed in Section 2.2.3. For this reduced dataset, with only 60% allocated for training the parameters of the optimizer were set to β_1_ = 0.5 and β_2_ = 0.999, and the learning rate at *lr* = 0.001. We then compared the results of the models trained with and without the low contrast images on both the entire test dataset and a subset of the test dataset that excluded low-contrast slices: results are presented in Table 3.

**Table 3:** Comparison of models trained with and without the slices with a Fisher's ratio < 0.05.

**Training contrast range**	**Test contrast range**	**Detection**	**Dice**	**HD**	**RAAD**
All (*n* = 14,415)	All (*n* = 245)	95 ± 1 (*)	0.65 ± 0.03	19 ± 2	−0.06 ± 0.04
*F*>0.05 (*n* = 8,836)	All (*n* = 245)	92 ± 3	0.65 ± 0.02	17 ± 1	−0.07 ± 0.03
All (*n* = 14,415)	*F*>0.05 (*n* = 173)	100 ± 0	0.72 ± 0.04	11 ± 2	0.65 ± 0.33
*F*>0.05 (*n* = 8,836)	*F*>0.05 (*n* = 173)	100 ± 0	0.73 ± 0.04 (*)	10 ± 2	0.68 ± 0.21

One-tailed paired Wilcoxon statistical test performed to verify if a model is significantly better than the other for the same test set (*** if *p* < 0.01, ** if *p* < 0.05, * if *p* < 0.09).

Excluding low-contrast slices from the training dataset had little impact on model performance when tested on the full test dataset, and marginally improved the DSC and HD when tested on slices with a Fisher's ratio >0.05. These results indicated that low-contrast slices (i.e., Fisher's ratio < 0.05) did not provide critical knowledge for segmentation. Thus, excluding low-contrast slices could allow for a significant reduction in computational time (from an average training time of 1 h and 50 min for the whole dataset to an average of 1 h and 20 min without the low contrast slices), without significant performance costs.

### 3.7 Transferability of the models

To ensure the reliability of our methodology, we extended our evaluation to both the RELATE and APIS datasets (see Section 2.1.2). Our goal was to investigate whether removing 40% of the training data would impact the transferability of our models to different datasets. We compared the performance of two models: one trained on the entire HIBISCUS-STROKE dataset and the other trained on the same dataset, excluding slices with a Fisher's ratio below 0.05.

Despite training the model without low-contrast slices on a reduced training dataset (40% less data), its performance on RELATE dataset was similar to the model trained on the complete dataset, as indicated in Table 4. Notably, both models achieved performance levels close to those shown in Table 3, even though the ground truth data and patient distribution differed significantly. These findings support the transferability of our model. Similarly, when tested on the APIS dataset, the models performed consistently irrespective of whether they were trained with all the slices or only those with higher contrast. However, it is important to note that the results were substantially poorer on the APIS dataset, as it contains acute-phase images, while our models were trained on subacute images. The task is particularly challenging in this context, as evidenced by the results from the APIS challenge, where participating teams achieved a Dice score of only around 0.20 when training on similar images.

**Table 4:** Comparison of models trained with and without the slices with a Fisher's ratio < 0.05 tested on RELATE and APIS datasets.

**Dataset**	**Training contrast range**	**Detection**	**Dice**	**HD**	**RAAD**
RELATE	All (*n* = 14,415)	98 ± 1 (*)	0.60 ± 0.01 (*)	22 ± 2	−0.17 ± 0.05 (**)
	*F*>0.05 (*n* = 8,836)	97 ± 1	0.60 ± 0.01	20 ± 0.5 (*)	−0.25 ± 0.02
APIS	All (*n* = 14,415)	98 ± 2	0.10 ± 0.01	55 ± 6 (**)	12.3 ± 0.7
	*F*>0.05 (*n* = 8,836)	99 ± 1 (*)	0.09 ± 0.01	70 ± 10	15.4 ± 7.6

One-tailed paired Mann–Whitney statistical test performed to verify if a model is significantly better than the other for the same test set (*** if *p* < 0.01, ** if *p* < 0.05, * if *p* < 0.09).

## 4 Discussion

In this study, we have developed a systematic method to assess how image contrast impacts the performance of a U-Net trained for stroke lesion segmentation on CT images. To validate our approach, we employed data augmentation techniques to standardize the training data in terms of image contrast variations and confirm that the unbalance of contrast across the dataset did not impact the performance for specific Fisher's ratio ranges. By analyzing the training outcomes using ROC curves and clustering techniques, we established a critical contrast threshold of 0.05. Below this threshold, the images no longer contribute meaningful information to the model. To confirm this finding, we trained a new model using a dataset that excluded low-contrast images, revealing opportunities to improve computational resource management during training.

Our study still presents certain limitations. CT imaging obtained at 24 h may underestimate the final lesion but has been routinely used in research studies as a surrogate of final stroke lesion (43), our use of baseline data based on FLAIR MRI mitigates biases in manual segmentation skills and ensures a representative sample of hospital cases. Despite these limitations, our approach demonstrated its effectiveness in stroke lesion segmentation on CT images. By implementing a contrast threshold of 0.05, we were able to remove ~40% of the training dataset while achieving nearly equivalent results compared to using the full dataset, even with low-contrast slices retained in the test set.

Although our methodology primarily focused on brain lesion segmentation, it can be adapted to other image segmentation tasks by identifying contrasts that yield the most efficient automatic segmentation. Indeed, experiments presented in the Supplementary material demonstrate improvements in performance with increased contrast for both stroke lesion segmentation using FLAIR MRI (Supplementary Section 2) and brain tumor segmentation using T1-weighted MRI (Supplementary Section 3). These findings suggest that it may be possible to define a contrast threshold to optimize computational efficiency by selecting only the most informative images for training. However, it is important to note that task-specific thresholds may need to be identified, as Fisher's ratio threshold of 0.05 is likely specific to stroke lesions on CT. To do so, some of the steps in the pipeline may need adaptation as the data augmentation considering the modality which can have different characteristics, considering the ranges of pixel values in the images and whether the object of interest appears hyper or hypodense among the rest of the tissue.

In our study, we used Fisher's ratio as a contrast measure, taking advantage of the presence of lesion reference and the adherence of the image modality (CT) to the Gaussian assumption and equal variances between the lesion and background. Depending on the algorithm and image modality, alternative metrics for assessing image contrast may be relevant. Furthermore, Fisher's ratio can be adjusted for other tasks by redefining what constitutes the background relative to the object of interest. For multiclass segmentation, the correlation between contrast levels of different regions may be considered. Future studies could investigate the impact of different preprocessing techniques on critical contrast and training performance.

The definition of this threshold can serve as an initial foundation to initiate discussions with clinicians, allowing them to gain insights into the complexity of the segmentation task and its implications. By distinguishing between informative and less informative contrast levels, clinicians gain a clearer understanding of how model performance varies across different image contrasts. This enhances their confidence in the model's segmentations, especially in high-contrast scenarios where performance remains robust beyond the threshold. Our pipeline automates the creation of a quality dataset, saving clinicians valuable time that they would normally spend on quality control. A more refined dataset not only improves confidence in model results but also model learning efficiency. Indeed, clinician feedback can be integrated into the validation stage, enabling continuous monitoring and refinement of the model's performance. Moreover, the definition of this contrast indicates that, when creating a new dataset, the focus can be placed on segmenting better-contrasted images without compromising the model's quality and not loosing time segmenting poorly-contrasted and difficult images. Ultimately, accurate segmentation on CT scans during the subacute phase will help assess the response to acute phase treatment, personalize management in the subacute phase, and refine prognostication. Furthermore, the identification of a contrast threshold opens avenues for improving overall model performance. Techniques such as curriculum learning (44) can be explored. This approach involves progressively presenting training data to the model based on their difficulty levels, which, in this context, could be determined by contrast variations.

## 5 Conclusion

In this study, we proposed an innovative methodology to evaluate the dataset quality in terms of contrast and its impact on stroke lesion segmentation with deep learning algorithms applied to CT images. We identified low-contrast images and excluded them from the training set through data analysis and visualization techniques. This approach significantly reduced computation time (by 30%) and resource requirements while maintaining segmentation performance for the remaining images. These experiments underscore the critical role of lesion contrast in training effective deep learning models for automatic segmentation, particularly with CT images. This work opens a dialogue with clinicians to explore the limitations, areas for improvement, and strategies for creating better datasets and training models. Although our methodology was specifically applied to stroke lesion segmentation in CT images, it could be adapted to other segmentation tasks by leveraging similar strategies and criteria.

## Data Availability

The data analyzed in this study is subject to the following licenses/restrictions. The anonymized data that support the findings of this study are available on request from the corresponding author. The data are not publicly available due to restrictions e.g., their containing information that could compromise the privacy of research participants. Requests to access these datasets should be directed to juliette.moreau@creatis.insa-lyon.fr.
