# Supplementary material for: Contrast quality control for segmentation task based on deep learning models—Application to stroke lesion in CT imaging
Source: Front Neurol. 2025 Feb 10;16:1434334. doi: 10.3389/fneur.2025.1434334 (PMC11849432; doi:10.3389/fneur.2025.1434334)
Supplement: Supplementary file 1 [file Data_Sheet_1.pdf]

frontiers

Supplementary Material

1 DATA AUGMENTATION ON CONTRAST ALGORITHM

Algorithm 1 Data contrast augmentation pseudo algorithm

```
slice                                                  ▷ the slice we are working on
mask                                                   ▷ its ground truth mask
bg_std_max, fisher_min, fisher_max                     ▷ characteristics depending on the task
x                                                      ▷ the HU intensity modification
nb                                                     ▷ number of steps to apply
for s : 0 : nb do                                      ▷ Method 1
    for i : 1 : height(mask) do
        for j : 1 : width(mask) do
            if mask(i,j) ≠ 0 and mask(i-1:i+1,j-1:j+1)> (x * s) then
                mask(i,j) ← mask(i,j)+x
            end if
        end for
    end for
    slice ← slice + mask
    if fisher(slice) > fisher_min and fisher(slice) < fisher_max then
        save slice
    end if
end for
if std(mask) > bg_std_max then                         ▷ Method 2
    for i : 1 : height(mask) do
        for j : 1 : width(mask) do
            if mask(i,j) ≠ 0 and mask(i,j)< median(mask) then
                mask(i,j) ← mask(i,j)+x
            end if
        end for
    end for
end if
slice ← slice + mask
if fisher(slice) > fisher_min and fisher(slice) < fisher_max then
    save slice
end if
```

2 IMPACT OF FISHER'S RATIO ON STROKE LESION SEGMENTATION IN FLAIR MRI

The HIBISCUS-STROKE dataset comprises FLAIR MRI images acquired 6 days post-stroke with segmented ground truth. Unlike CT scans, contrast is not a significant issue in this modality, and the Fisher's ratio distribution is more uniform (Figure S1). However, using a model trained on 80% of the dataset, with 10% for validation and 10% for testing (randomly selected), performance improves with higher contrast (Figure S2). This indicates that applying our proposed methodology could help identify a contrast threshold and enable more efficient dataset selection, even if contrast is not a primary concern in this case.

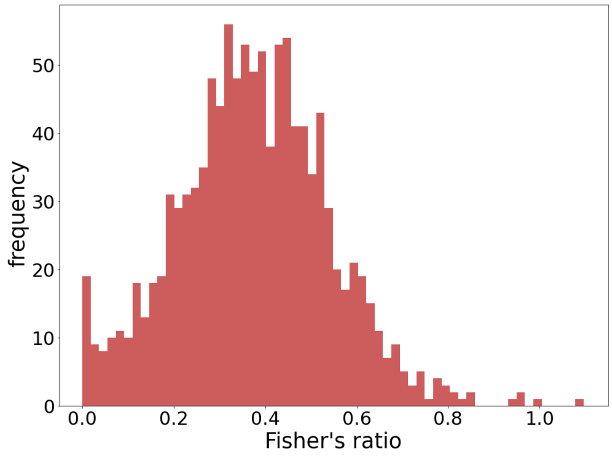

Figure S1. Distribution of Fisher's ratio in the HIBISCUS-STROKE FLAIR MRI dataset.

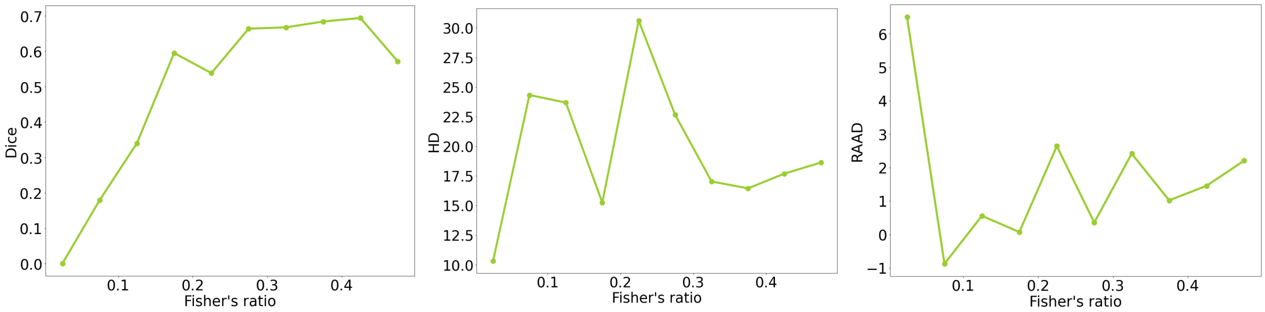

Figure S2. Preliminary experiments on the HIBISCUS-STROKE FLAIR MRI dataset for stroke lesion segmentation. Metrics evolution (Dice, HD, and RAAD) with increasing contrast in the test set.

3 IMPACT OF FISHER'S RATIO ON TUMOR SEGMENTATION IN T1 MRI

The experiment described in Appendix 2 was repeated using the BRATS (Brain Tumor Segmentation Challenge) dataset Menze et al. (2014), which contains multimodal brain MRI scans with tumor annotations and corresponding segmentation masks. For this experiment, we selected and preprocessed hundred 3D volumes similarly to the stroke images, focusing solely on the T1 MRI sequence. To simplify the task, we grouped the three tumor labels into a unique binary segmentation framework. As with the stroke CT scans, the distribution of Fisher's ratio across slices (Figure S3) is highly uneven, even though tumor contrast is not typically regarded as a significant challenge. This unexpected distribution arises from merging the tumor core, peritumoral tissue, and enhancing tumor regions, which are inherently heterogeneous. Consequently, the variance of intensities within the tumor region — used as the denominator in Fisher's ratio calculation — becomes large, resulting in very small ratio values despite the tumor being visually distinct from surrounding brain tissue. Segmentation performance improve with increased contrast in the test set (Figures S4), except for RAAD, which exhibits a different trend. This observation suggests that our proposed methodology could help identify an optimal contrast threshold, potentially improving training efficiency.

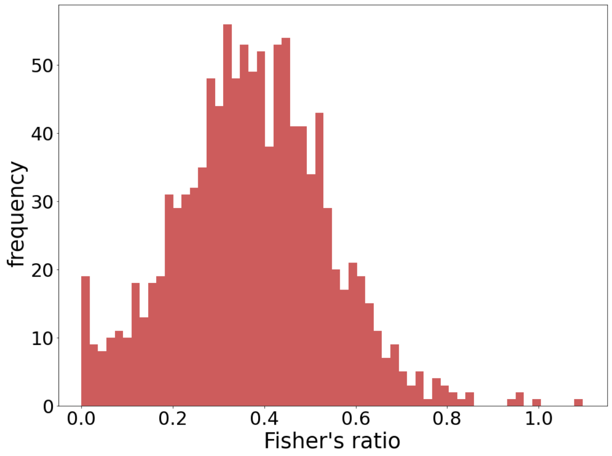

Figure S3. Distribution of Fisher's ratio in the HIBISCUS-STROKE FLAIR MRI dataset.

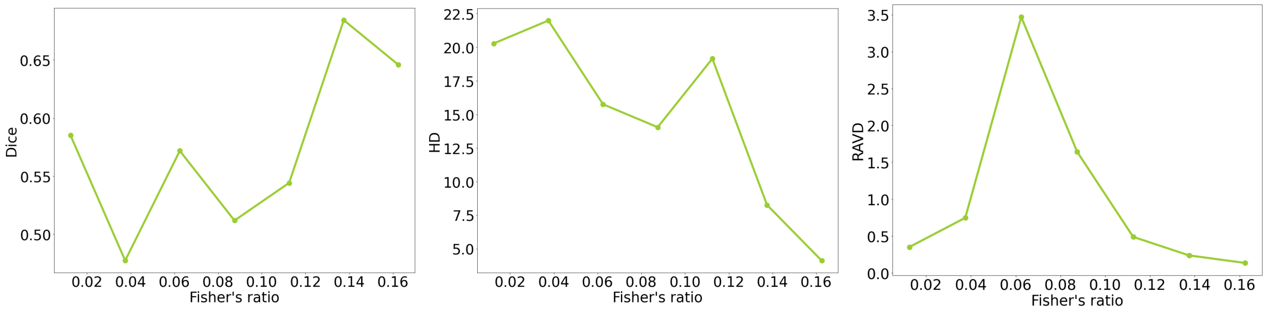

Figure S4. Preliminary experiments on the BRATS T1 MRI dataset for tumor lesion segmentation. Metrics evolution (Dice, HD, and RAAD) with increasing contrast in the test set.

REFERENCES

Menze, B. H., Jakab, A., Bauer, S., Kalpathy-Cramer, J., Farahani, K., Kirby, J., et al. (2014). The multimodal brain tumor image segmentation benchmark (brats). *IEEE transactions on medical imaging* 34, 1993–2024
